# SB431542 partially inhibits high glucose-induced EMT by restoring mitochondrial homeostasis in RPE cells

**DOI:** 10.1186/s12964-023-01372-1

**Published:** 2024-01-05

**Authors:** Jingjing Cao, Mingfei Jiao, Zhenyu Kou, Feifei Han, Lijie Dong

**Affiliations:** Tianjin Key Laboratory of Retinal Functions and Diseases, Tianjin Branch of National Clinical Research Center for Ocular Disease, Eye Institute and School of Optometry, Tianjin Medical University Eye Hospital, Tianjin Medical University Eye Institute, 251 Fukang Road, Nankai, Tianjin, 300384 P.R. China

**Keywords:** SB431542, RPE, Proliferative diabetes retinopathy, Epithelial mesenchymal transformation, Mitochondria

## Abstract

**Background:**

The epithelial–mesenchymal transition (EMT) of retinal pigment epithelial (RPE) cells participated in the development of retinal fibrosis. SB431542 is a small molecule inhibitor with inhibitory effects on the ALK4, ALK5 and ALK7. Our study aimed to explore the effect of SB431542 on the EMT of RPE cells and to provide new ideas for the treatment of retinal fibrosis.

**Methods:**

We performed fundus fluorescein angiography, optical coherence tomography and hematoxylin–eosin staining in vivo to observe the effect of SB431542 on choroidal neovascularization (CNV)-induced retinopathy. The proliferation, migration, cytoskeleton, adhesion, reactive oxygen species (ROS), mitochondrial morphology and membrane potential of RPE cells were observed in vitro through fluorescein diacetate staining, Cell Counting Kit-8 experiment, wound healing assay, phalloidin staining, immunofluorescence, MitoSOX, DCFH-DA, MitoTracker and JC-10 staining. Western blot, reverse transcription quantitative and immunofluorescence were used to detect the expression of EMT–related markers, pERK1/2, pGSK3β and β-catenin.

**Results:**

SB431542 significantly alleviated retinopathy in the CNV model. The proliferation, migration and adhesion in RPE cells decreased to a certain extent in SB431542 treatment. SB431542 partially normalized the structure of RPE cells. The expression levels of E-cadherin increased, while the expression levels of laminin and N-cadherin decreased with SB431542 treatment. SB431542 reduced the production of total ROS, mitochondrial SOX and recovered the mitochondrial membrane potential to a certain degree. In addition, our study showed that SB431542 downregulated the phosphorylation of ERK1/2, GSK3β and the expression of β-catenin.

**Conclusion:**

SB431542 improved EMT in RPE cells by maintaining mitochondrial homeostasis via the ERK1/2 and GSK3β/β-catenin pathways.

Video Abstract

**Graphical Abstract:**

SB431542 inhibits EMT in RPE cells under high glucose conditions.

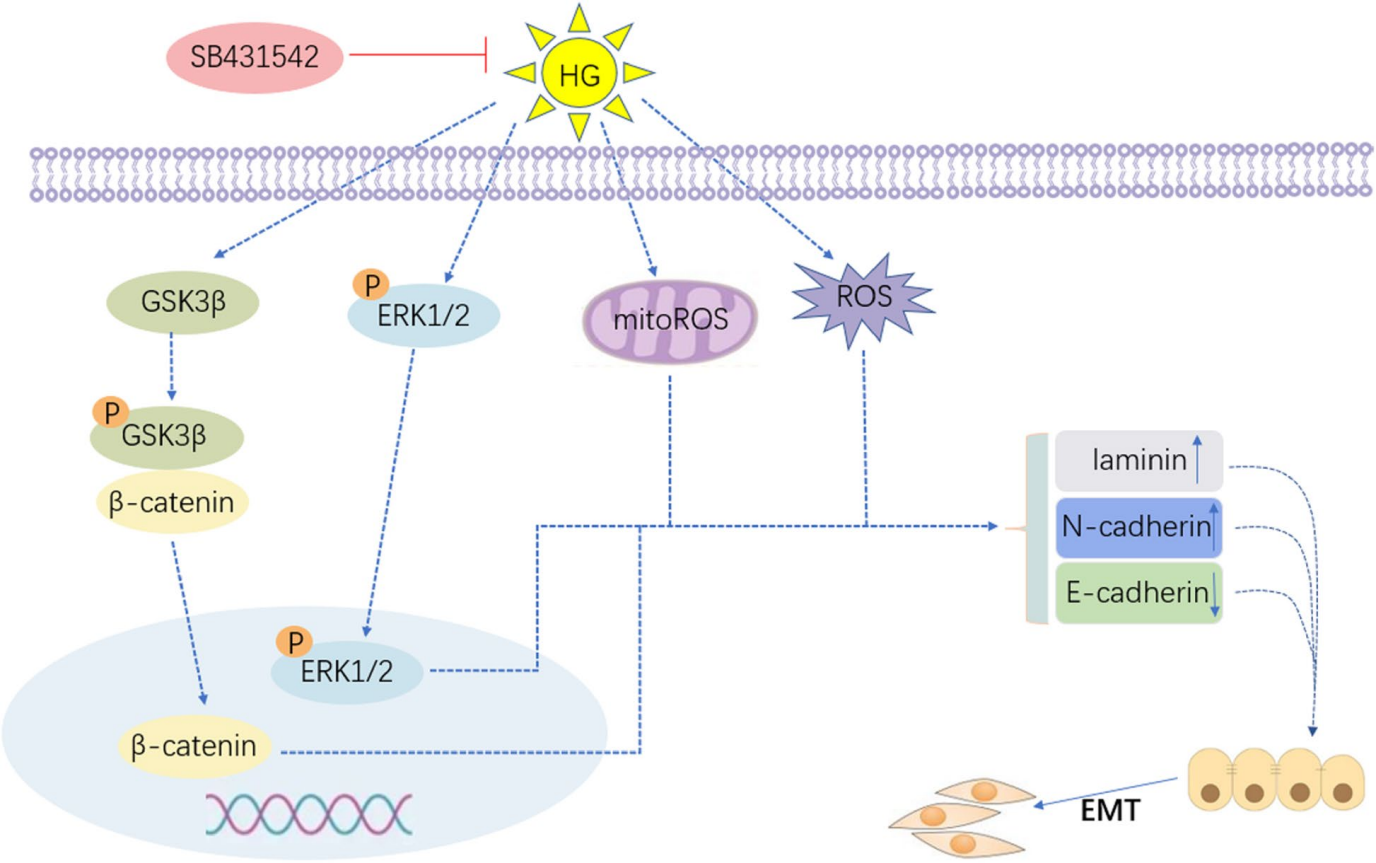

**Supplementary Information:**

The online version contains supplementary material available at 10.1186/s12964-023-01372-1.

## Introduction

Diabetic choroidal lesion refers to the abnormal changes in various choroidal microvascular structures secondary to diabetes mellitus (DM), ultimately leading to choroidal neovascularization (CNV) and fibrous membrane proliferation [1]. Retinal fibrosis in the later stage of CNV caused irreversible damage to diabetic retinopathy (DR) vision [2]. Therefore, targeting subretinal fibrosis has become a new method for treating DR.

Retinal pigment epithelial (RPE) cells are the primary cells that cause the formation of fibrous proliferative membranes [3]. The occurrence of fibrosis is mostly due to the epithelial–mesenchymal transition (EMT) of RPE cells, which then differentiate into fibroblasts. The proliferation and migration of RPE cells lead to the formation of fibrous proliferative membranes [4]. Therefore, we aim to treat subretinal fibrosis by reversing the EMT of RPE cells.

The normal energy metabolism of cells was largely maintained through the oxidative phosphorylation of mitochondria [5]. Mitochondrial dysfunction triggers the production of reactive oxygen species (ROS), exacerbating the progression of EMT [6]. Sufficient evidence suggests that impaired mitochondrial function and weakened mitochondrial metabolism are associated with the development of EMT and involved in the progression of hepatocellular carcinoma [7]. Research found that mitochondrial homeostasis was closely related to EMT in endometrial cancer cells [8]. However, the role of mitochondria in the EMT of RPE cells is currently unknown. Therefore, the objective of our study is to elucidate the effects of mitochondrial homeostasis on EMT in RPE cells.

SB431542 is TGF-β receptor kinase inhibitor with inhibitory effects on the activities of ALK4, ALK5 and ALK7. SB431542 has been proven to disturb TGF-β signal transduction to alleviate liver fibrosis and to block TGF-β/Smad2/3 signal transduction to prevent pulmonary fibrosis [9, 10]. To date, limited research has been conducted on SB431542 in the field of retinal fibrosis. One study found that SB431542 suppressed the differentiation of RPE cells induced by the TGF-β pathway [11]. Whether SB431542 may play a role through other mechanisms in RPE cells remains to be studied.

Our study elucidated the effect of SB431542 on the EMT of RPE cells under high glucose conditions. In addition, we detected possible mechanisms by which SB431542 may play a role.

## Materials and methods

### Animals

C57/BL6J mice were purchased from Vital River (Beijing, China). All procedures complied with the international ethical use guidelines of experimental animals. This experiment was approved by the Medical Experimental Animal Ethics Committee of Tianjin Medical University Eye Hospital (TJYY20190103002). First, 24 mice were randomly divided into 4 groups: normal, CNV, CNV+DMSO and CNV+SB431542 groups (10mg/kg, continuous intraperitoneal injection for 5 days, 1 time per day, MCE Corporation, HY-10431).

### Establishment of CNV model

Argon laser photocoagulation (532 nm wavelength, 100 μm spot size, 0.05 duration, and 100 mW) was employed to generate four to six laser spots in each eye surrounding the optic nerve. The production of a vaporization bubble at the laser spot indicated the rupture of Bruch’s membranle, which is an important factor in successful CNV. The mice were anesthetized with an intraperitoneal injection of 4% chloral hydrate (Aladdin, C104202) at a concentration of 10mL/kg, while compound tropicamide eye drops (Zhuobian, H20055546) were added for mydriasis (Phoenix Research Labs, USA). In addition, we injected the mice intraperitoneally with 5% sodium fluorescein (Akorn, Decatur, IL, USA; 50μL/mouse). Retinopathy in mice were observed with OCT (OCT; Spectralis; Heidelberg Engineering, Inc., Carlsbad, CA, USA). The area of the retinopathy was quantified via Image J.

### Hematoxylin–eosin (HE)staining

To further evaluate the pathological changes of CNV, paraffin sections were prepared for HE staining. The area of the retinopathy was quantified via Image J.

### Cell culture

Human RPE cell line (ARPE-19 cells) was purchased from the Guangzhou Geneo Biological Department Technology Company. The cells were divided into five groups: N group (normal medium containing 5.5mM glucose), M group (25mM mannitol), HG group (25mM glucose), HG+DMSO group (5μM DMSO), HG+SB431542 group (5μM SB431542 dissolved in DMSO acted on cells for 48 h). The cells were cultured for 48 h for subsequent experiments.

### Cell viability assay

The cells were inoculated into a 96-well plate and then the corresponding treatment was performed. The medium was then replaced with fresh medium mixed with Cell Counting Kit-8 (CCK-8) reagents (CA1210, Solarbio). After incubation for 2 h, absorbance at 450 nm was measured with an acid standard instrument (INFINITE M200).

### Wound healing assay

The cells were inoculated into a 6-well plate and cultured until confluence. A linear wound was created by vertically scraping the cells. Migration was recorded under a microscope (BX51T-32FB-E01) at 0h and 12h after scratching. Image J was used to measure cell migration.

### Fluorescein diacetate (FDA) staining

The cells were inoculated into a 48-well plate and then incubated in 10μM FDA (Solarbio, IF0160) at 37°C for 20 min. After washing three times with a serum-free medium, the nuclei were stained with Hoechst for 1 min. Fluorescence microscope photographs were taken via fluorescence microscopy (BX51T-32FB-E01) and fluorescence intensity was quantitatively analyzed using Image J.

### Immunofluorescence

Cells were fixed in 4% PFA for 10 min, permeabilized in 0.1% Triton X-100 (Solarbio, T8200) for 10 minutes and then incubated in a solution that consisted of 1% BSA (Solarbio, SW3015) and 0.1% Tween (Solarbio, T8220) for 30 min. After the corresponding primary antibody (pERK1/2, Affinity, AF1015,1:300; pGSK3β, Affnity, AF2016,1:300; β-catenin, Affnity, AF6266,1:300; vinculin, Affnity, AF5122,1:300) were incubated for overnight at 4°C, secondary antibody (Abbkine, A23220) was incubated at room temperature for 1h. The distribution of proteins was observed under fluorescence microscopy (BX51T-32FB-E01) and fluorescence intensity was quantitatively analyzed using Image J.

### Western blot (WB)

The protein lysates were separated on SDS-PAGE gel and then transferred to polyvinylidene difluoride (PVDF) membranes. The blots were incubated for overnight with the primary antibodies including nodal (Affinity, DF7791,1:1000), laminin (abcam, ab11575,1:5000), N-cadherin (Affinity, AF5239,1:1000), E-cadherin (Affinity, AF0131,1:1000), pERK1/2 (Affinity, AF1015,1:1000), ERK1/2(Affinity, AF0155,1:1000), pGSK3β (Affnity, AF2016,1:1000), GSK3β (Affnity, AF5016,1:1000), β-catenin(Affnity, AF6266,1:1000), β-actin (Affinity, AF7018,1:1000). After washing with TBST, goat anti-rabbit (H+L) HRP (Affinity, S0001, 1:3000) was incubated at room temperature for 2h. The membrane was visualized using the Omni-ECL™™Femto Light Chemilumcence Kit (EpiZyme, SQ201).

### Reverse transcription quantitative polymerase chain reaction (RT-qPCR)

Total RNA was extracted from RPE cells using the EZ-press RNA Purification Kit (EZBioscience, B0004DP). RNA was reversely transcribed to complementary DNA (cDNA) by using SweScript RT I First Strand cDNA Synthesis Kit (Servicebio, G3330-100). The RT-qPCR reactions were performed using 2× SYBR Green qPCR Master Mix (Servicebio, G3322-05). The primers used in the process are provided in Table 1.

**Table 1:** List of primers

Gene	Primer sequence (5'-3')
β-actin	F: CCTGGCACCCAGCACAAT
	R: GGGCCGGACTCGTCATAC
Nodal	F: GCTCCTTATGCTCTACTCCA
	R: GAACTTGACCTTCCGACA
Laminin	F: GAAGACGGGAAGAAAGGG
	R: TGCAAGTGGCTGACGATA
N-cadherin	F: ATCCTACTGGACGGTTCG
	R: TTGGCTAATGGCACTTGA
E-cadherin	F: CCCCATACCAGAACCTCG
	R: TGTGCCTTCCTACAGACG

*F* Forward, *R* Reverse

### Detection of ROS and mitochondrial SOX

DCFH-DA (Beyotime, S0033S) was used to detect total ROS and MitoSOX red mitochondrial superoxide indicator (Yeason, 40778ES50) was used to detect mitochondrial SOX. Cells were stained with 10µM DCFH-DA (Beyotime, S0033S) and 5 µM MitoSOX red mitochondrial superoxide indicator (Yeason, 40778ES50) working solutions respectively. After washing three times with a serum-free medium, the nuclei were stained with Hoechst for 1 min and images were obtained via fluorescence microscopy (BX51T-32FB-E01). Fluorescence intensity was quantitatively analyzed using Image J.

### Mito-Tracker Red CMXRos staining

Mito-Tracker Red CMXRos (Beyotime, C1049B) can specifically label active mitochondria. The cells were incubated in 100nM working solution at 37 °C for 20 min. Fresh culture medium was added after removing the staining solution. Images were captured via fluorescence microscopy (BX51T-32FB-E01) and fluorescence intensity was quantitatively analyzed via Image J.

### JC-10 staining

The cells were seeded in 48-well plates and then incubated with 20µM JC-10 solution (Yeasen, 40707ES03) in the buffer for 30 min. Fluorescence expression was observed via fluorescence microscopy (BX51T-32FB-E01) and quantitatively analyzed using Image J.

### Statistical analysis

All experiments were performed at least three times. Quantitative data were presented as mean ± standard deviation and the one-way ANOVA was conducted to analyze data difference. *P<*0.05 was considered statistically significant.

## Results

### SB431542 reduced retinopathy in CNV models

**Fig. 1:**
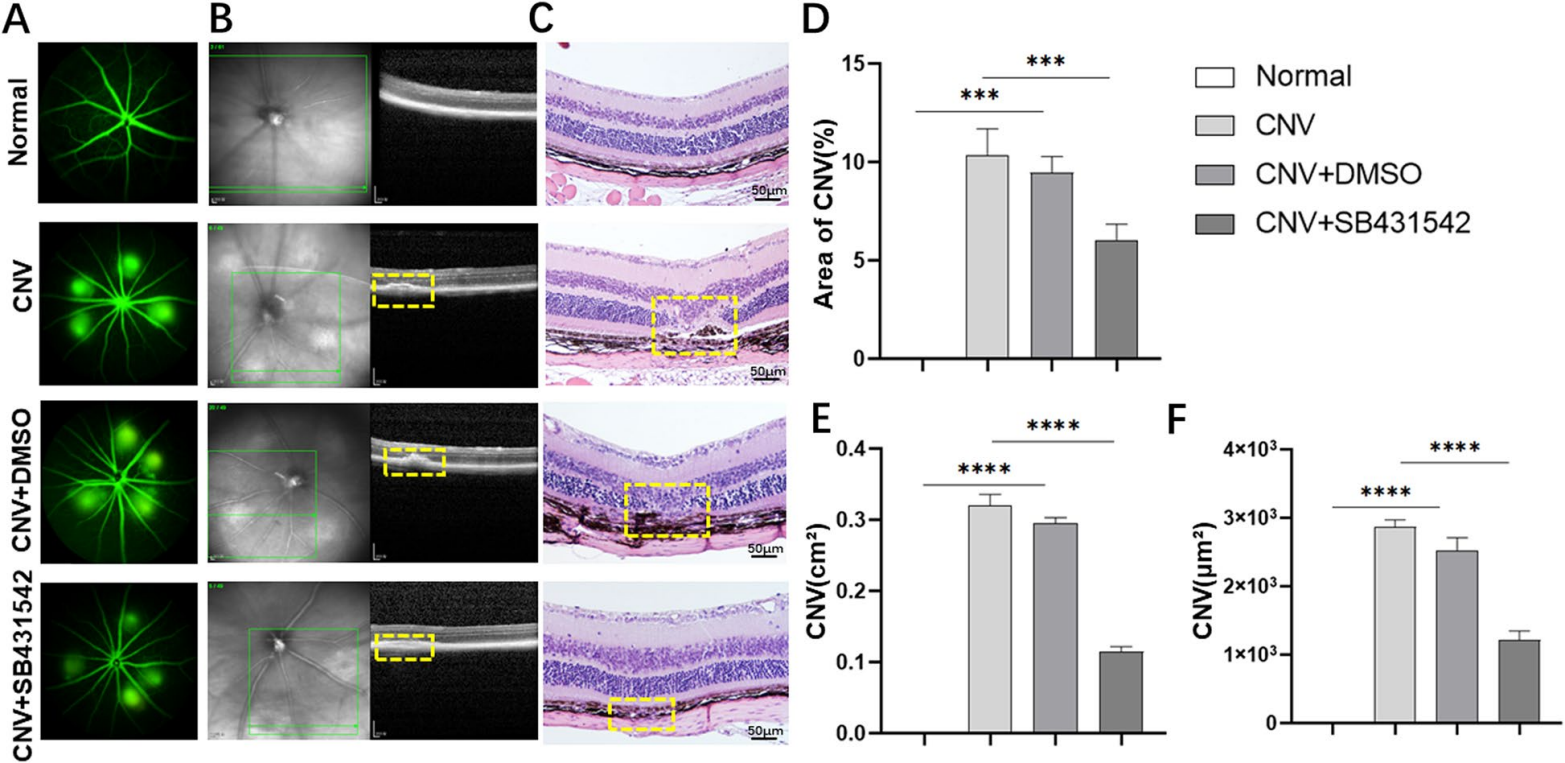
Effects of SB431542 on retinopathy in CNV models. **A**, **D** FFA observation fluorescein leakage and quantitative analysis. **B**, **E** Observation of retinopathy area via OCT and quantitative analysis. **C**, **F** Observation of retinopathy area via HE staining and quantitative analysis. ****P<0.0001, ***P<0.001

The effects of SB431542 on CNV in vivo were observed through FFA, OCT and HE staining. The area of fluorescein leakage in the CNV+SB431542 group was significantly restored compared with those in the CNV and CNV+DMSO groups (Fig. 1A and D). Both OCT (Fig. 1B and E) and HE staining (Fig. 1C and F) showed that the RPE layer outside the retina was damaged, also causing destruction to the internal structure of the retina when the outer barrier was impaired (within the yellow square frame). The area of retinopathy in the CNV+SB431542 group was significantly reduced compared with those in the CNV and CNV+DMSO groups. These results showed that SB431542 reduced retinopathy in CNV models.

### SB435142 partially inhibited the proliferation and migration of RPE cells induced by high glucose

**Fig. 2:**
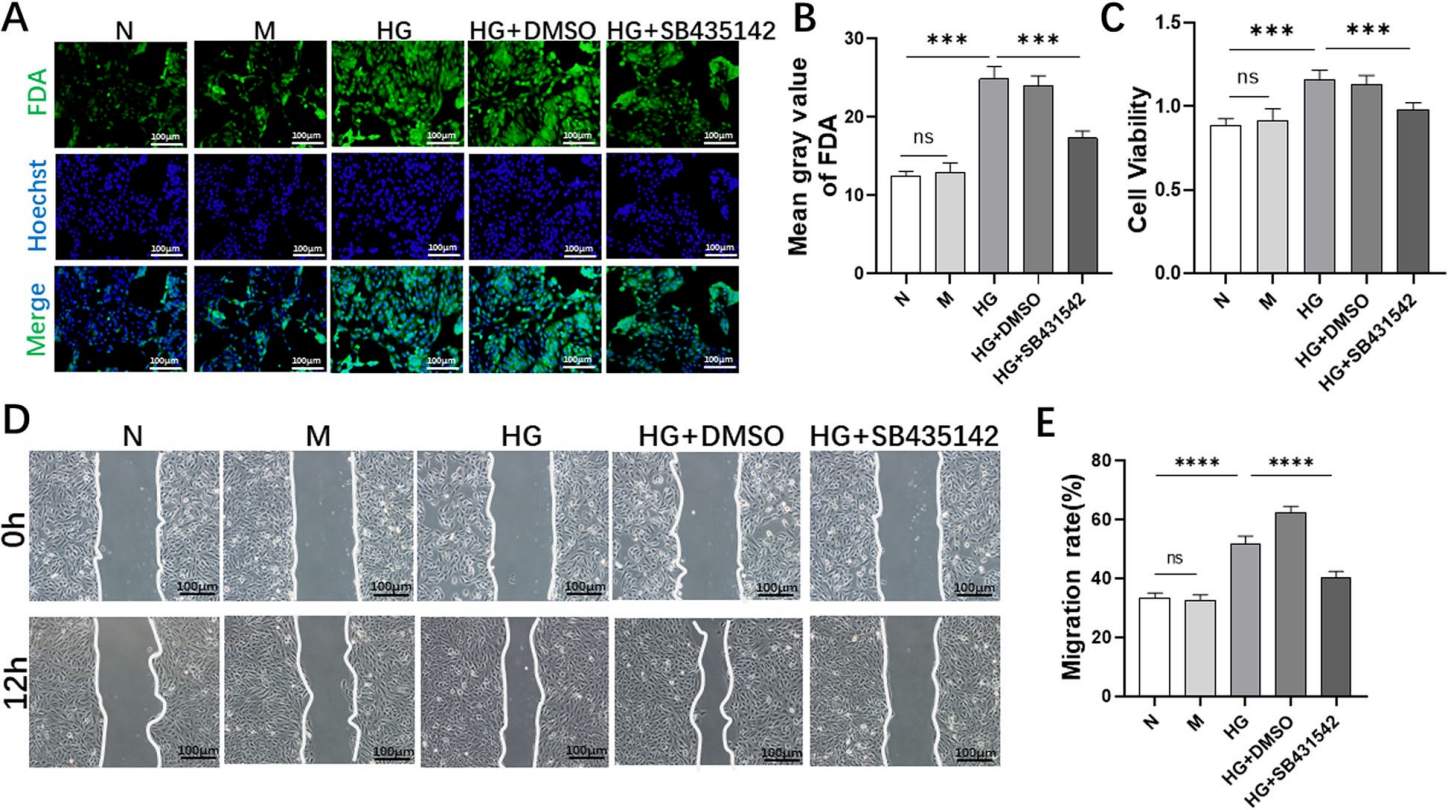
Effects of SB431542 on the proliferation and migration of RPE cells. **A-B** FDA staining detected cell density and quantitative analysis. (C)CCK-8 detected cell viability. **D-E** Wound healing assay detected the cell migration rate and quantitative analysis. ****P<0.0001, ***P<0.001

The proliferation and migration of cells promoted the occurrence of EMT. Therefore, we tested the effects of SB431542 on the proliferation and migration of RPE cells through FDA staining (Fig. 2A and B), CCK-8 (Fig. 2C) and wound healing assay (Fig. 2D, E). We found that the proliferation and migration abilities of RPE cells in the HG group were enhanced. Meanwhile, after treatment with SB431542, the proliferation and migration abilities of RPE cells in the HG+SB431542 group were weakened. Therefore, we concluded that SB431542 inhibit the proliferation and migration of RPE cells to a certain extent under high glucose conditions.

### SB431542 partially normalized the cytoskeleton and the adhesion of RPE cells induced by high glucose

**Fig. 3:**
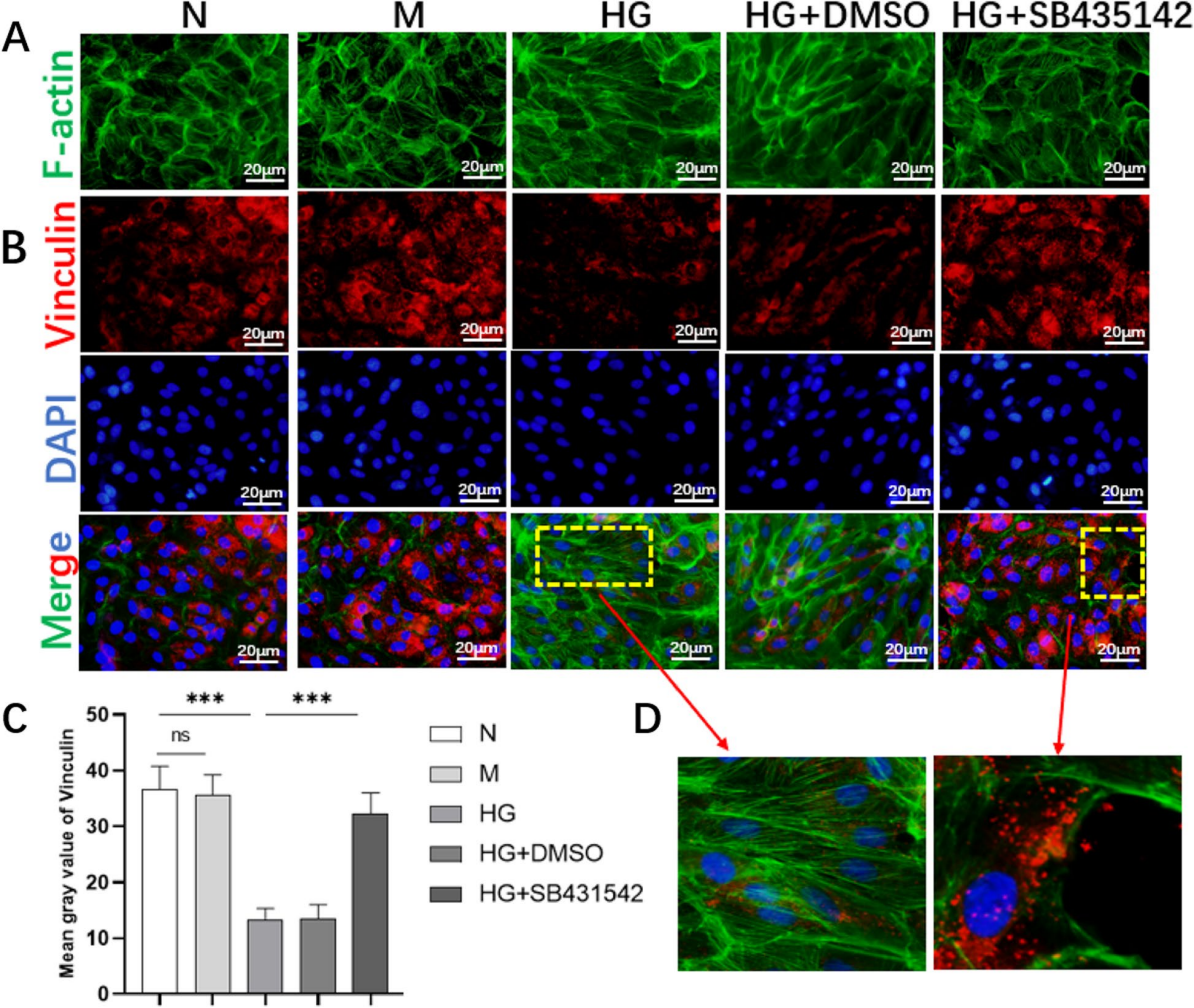
Effects of SB431542 on the cytoskeleton and adhesion of RPE cells. **A** Phalloidin stained cytoskeleton protein F-actin for evaluating the cytoskeleton. **B** Expression of adhesive protein vinculin was evaluated via immunofluorescence. **C** Fluorescence intensity of vinculin was quantitatively detected. **D** Enlarged images of the cytoskeleton protein F-actin and adhesive protein vinculin. ***P<0.001

The cells tended to be fibrotic-like and the expression of the adhesion protein vinculin reduced in cells induced by high glucose. After treatment with SB431542, the structure of cells returned to normalization (Fig. 3A and D) and cell adhesion was also enhanced (Fig. 3B-D) in the HG+SB431542 group. Therefore, SB431542 partially normalized the cytoskeleton and the adhesion of RPE cells.

### SB431542 affected the expression of EMT-related markers induced by high glucose

**Fig. 4:**
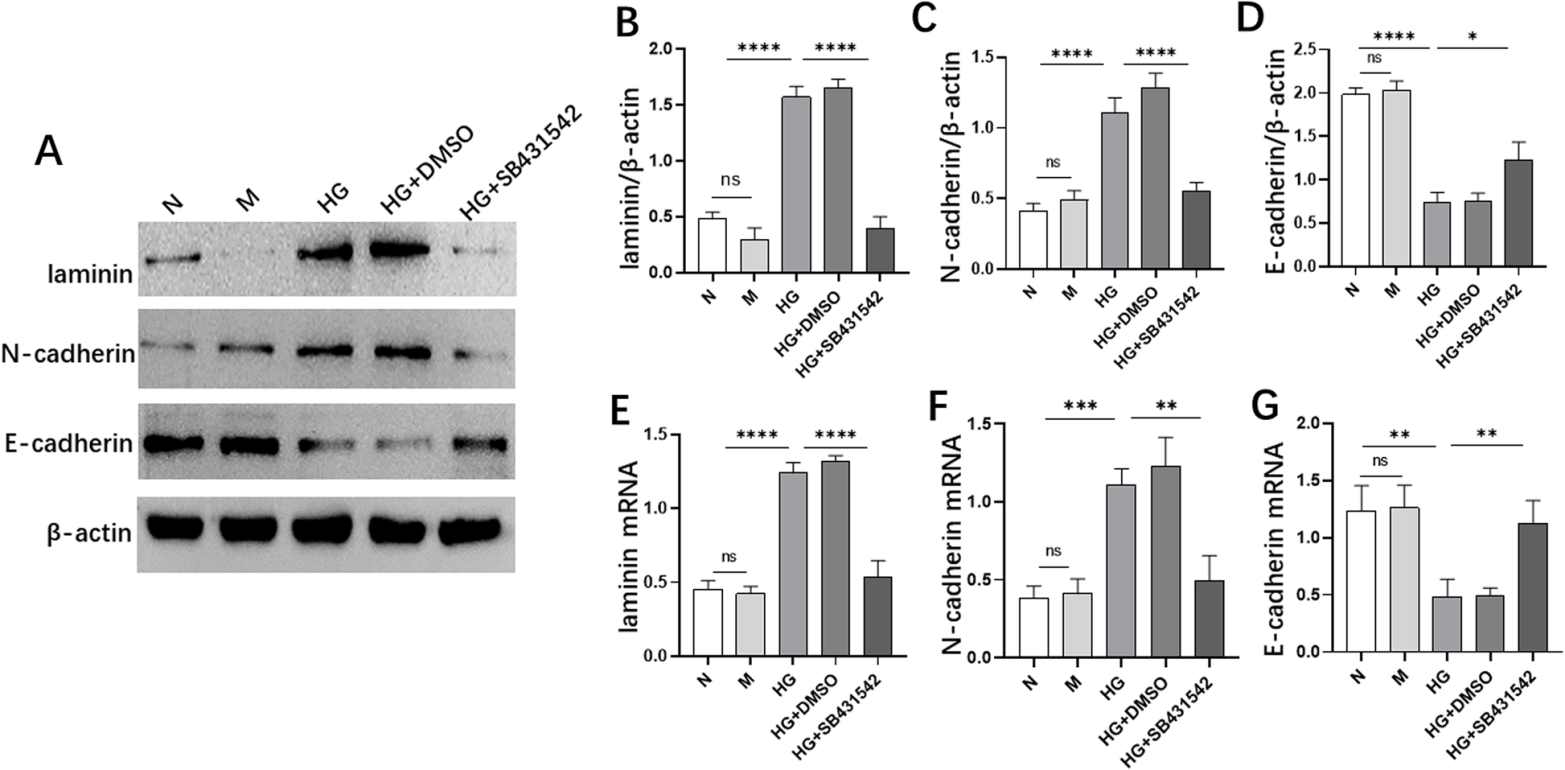
Effects of SB431542 on the expression of EMT-related markers. **A** WB detected the expression levels of laminin, N-cadherin and E-cadherin. **B** Quantitative protein expression of laminin. **C** Quantitative protein levels of N-cadherin. **D** Quantitative protein levels of E-cadherin. **E** The mRNA level of laminin was detected via RT-qPCR. **F** The mRNA level of N-cadherin was detected via RT-qPCR. **G** The mRNA level of E-cadherin was detected via RT-qPCR. ****P<0.0001, ***P<0.001, **P<0.01, *P<0.05

Our study observed the expression of mesenchymal markers (laminin and N-cadherin) and epithelial markers (E-cadherin). The expression levels of mesenchymal markers laminin (Fig. 4B and E) and N-cadherin (Fig. 4C, F) were significantly reduced in the HG+SB431542 group compared with those in HG+DMSO group. Meanwhile, the level of epithelial markers E-cadherin (Fig. 4D and G) was significantly increased via WB (Fig. 4A) and RT-qPCR. These results indicated that SB431542 partially inhibited EMT induced by high glucose.

### SB431542 partially inhibited high glucose-induced mitochondrial dysfunction in RPE cells

**Fig. 5:**
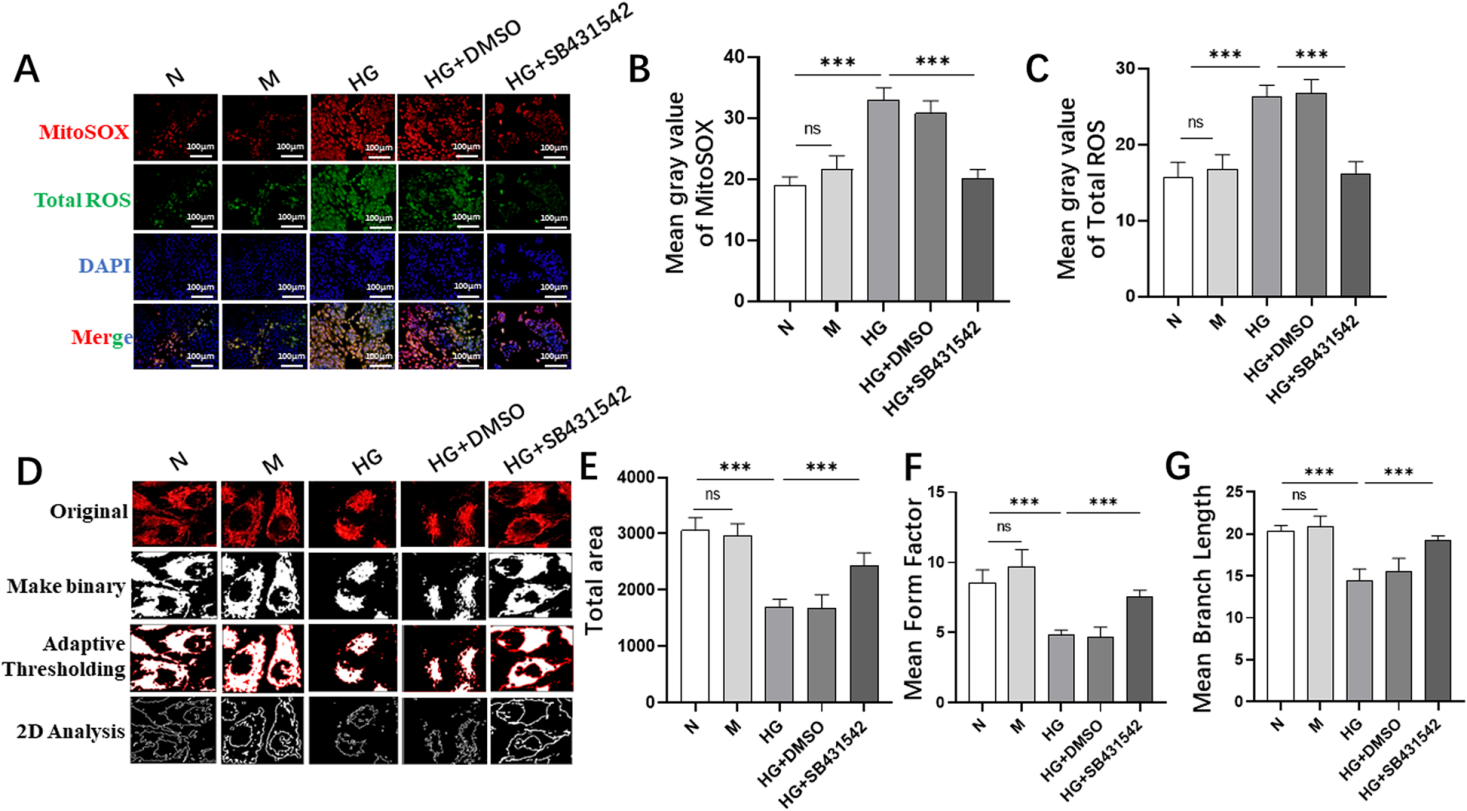
Effect of SB431542 on mitochondrial dysfunction induced by high glucose. **A** Comparing the levels of mitochondrial ROS and total ROS in each group by using MitoSOX and DCFH-DA staining. **B** Quantitative detection of mitochondrial ROS levels. **C** Quantitative detection of total ROS levels. **D** Mito-Tracker Red CMXRos was used to evaluate mitochondrial morphological changes. **E** Quantitative detection of total area. **F** Quantitative detection of mean form factor. **G** Quantitative detection of mean branch length. ***P<0.001

The levels of mitochondrial SOX and total ROS in each group were tracked by using MitoSOX and DCFH-DA staining (Fig. 5A), the levels of mitochondrial SOX (Fig. 5B) and total ROS (Fig. 5C) in the HG+SB431542 group were determined to be lower than those in the HG+DMSO group. Moreover, we stained mitochondria with Mito-Tracker (Fig. 5D) and evaluated mitochondrial morphology with mitochondrial total area (Fig. 5E), mean form factor (Fig. 5F) and branches (Fig. 5G). Mitochondrial total area, mean form factor and branches in the HG+SB431542 group were significantly higher than those in the HG group. In summary, SB431542 protected mitochondria from high glucose damage.

### SB431542 promoted the recovery of mitochondrial membrane potential in RPE cells induced by high glucose

**Fig. 6:**
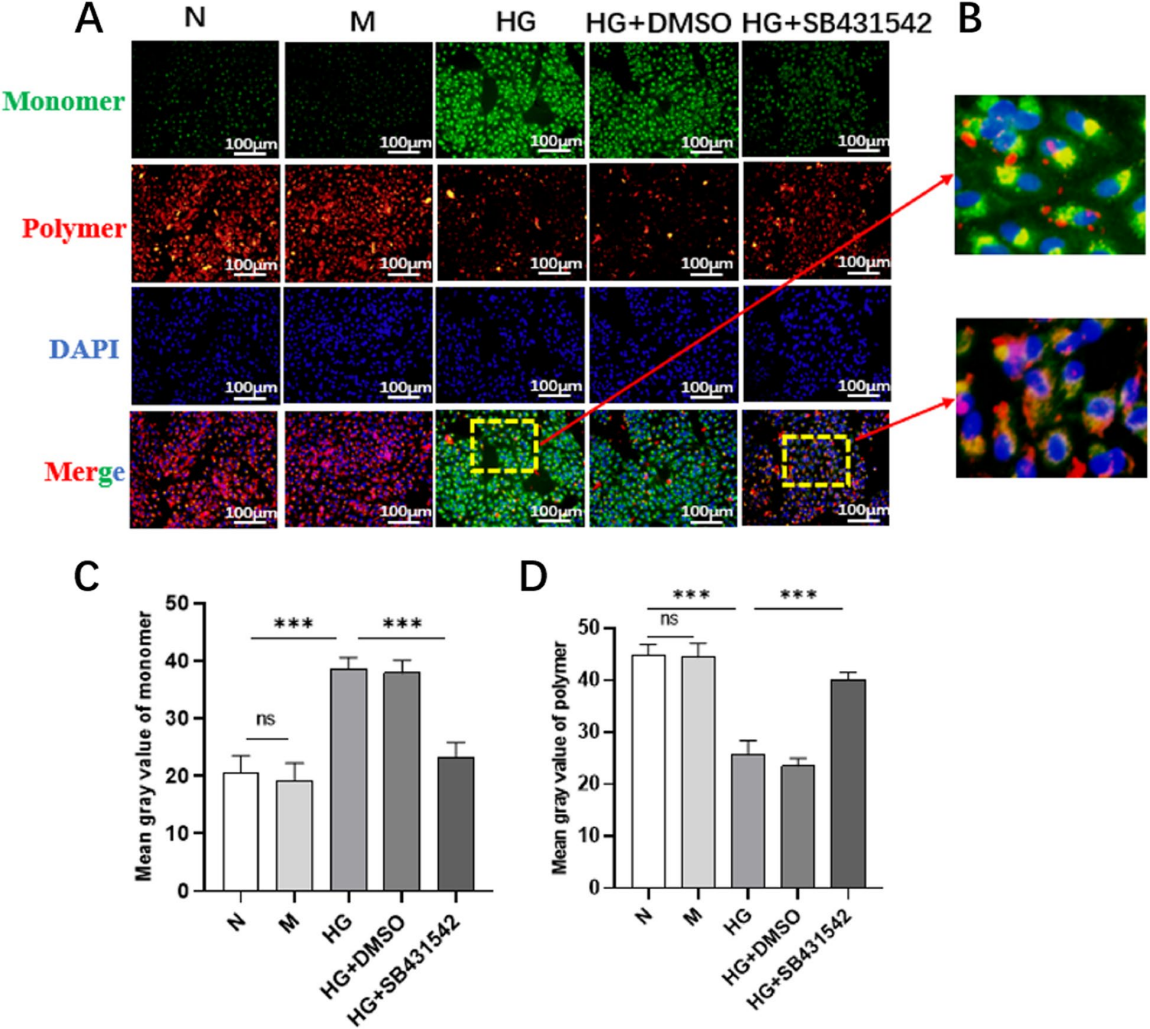
Effect of SB431542 on the mitochondrial dysfunction induced by high glucose. **A** Detection of mitochondrial membrane potential via JC-10 staining. **B** Enlarged images of JC-10 staining in high glucose and high glucose+SB431542 groups. **C** Quantitative detection of monomer production. **D** Quantitative detection of polymer production. ***P<0.001

In addition, the changes in mitochondrial membrane potential were assessed via JC-10 staining (Fig. 6A). The level of polymers in the HG+SB431542 group was higher than that in the HG+DMSO group (Fig. 6C), while the level of monomers was lower (Fig. 6D). The results indicated that SB431542 restored mitochondrial membrane potential to a certain degree.

### SB431542 blocked the phosphorylation of ERK1/2 extracellular signal-regulated kinase (ERK1/2) in RPE cells

**Fig. 7:**
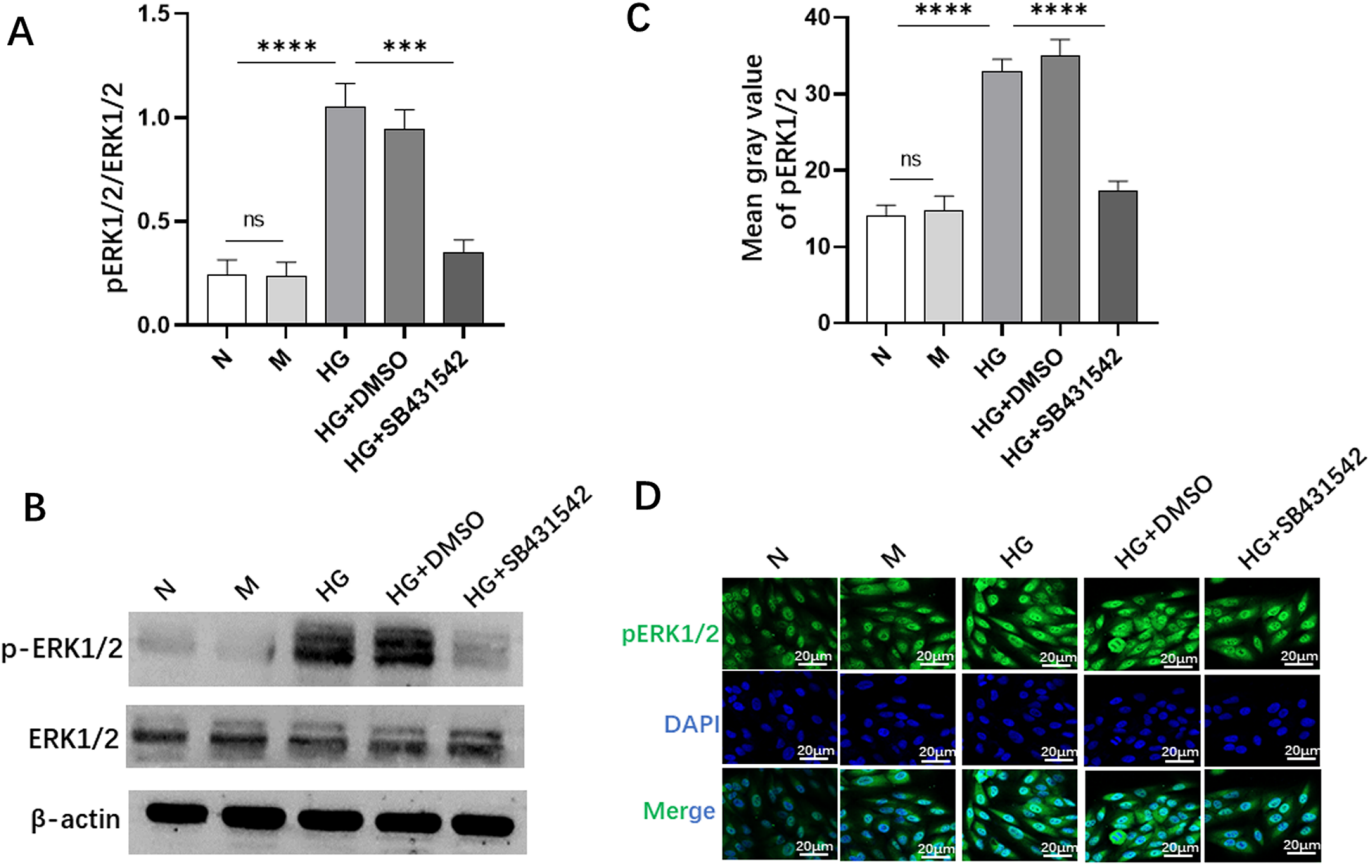
Effect of SB431542 on the expression of ERK1/2 activity. **A** Quantitative protein levels of pERK1/2. **B** WB detection of the expression of ERK1/2 activity. **C** Quantitative fluorescence intensity of pERK1/2. **D** Immunofluorescence detection of the expression of pERK1/2. ****P<0.0001, ***P<0.001

Our study observed the effect of SB431542 on ERK1/2 via WB (Fig. 7B) and found that the phosphorylation level of ERK1/2 (Fig. 7A) in the HG+SB431542 group was lower than that in the HG+DMSO group. The levels of pERK1/2 (Fig. 7C, D) also decreased in the HG+SB431542 group as determined by immunofluorescence. The results indicated that SB431542 may inhibit EMT by inhibiting the phosphorylation of ERK1/2.

### SB431542 prevented the phosphorylation of glycogen synthase kinase β (GSK3β) and the expression of β-catenin in RPE cells

**Fig. 8:**
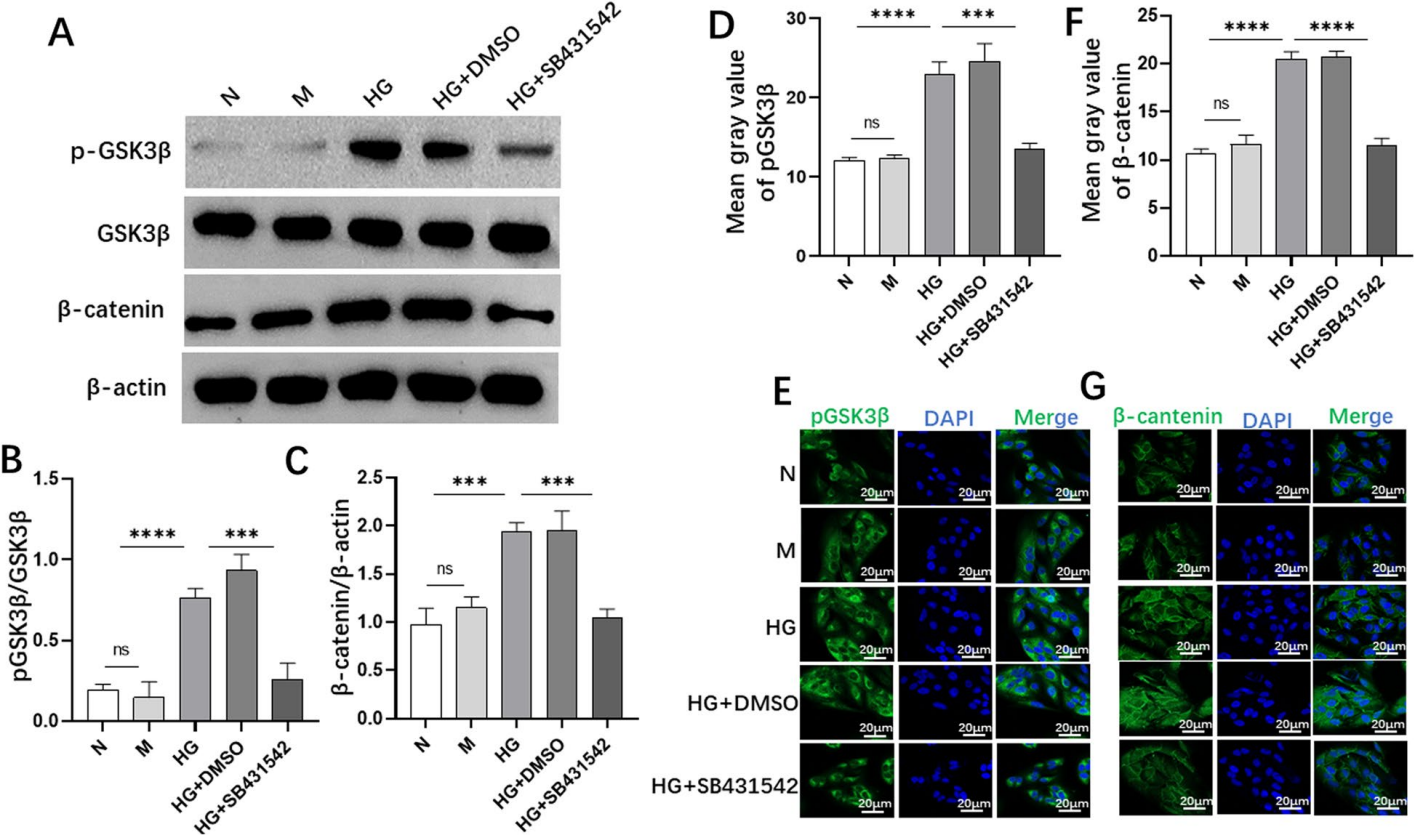
Effects of SB431542 on the expression of pGSK3β and β-catenin induced by high glucose. **A** WB detection of the expression of pGSK3β and β-catenin. **B** Quantitative analysis of the protein levels of pGSK3. **C** Quantitative detection of the protein levels of β-catenin. **D** Quantitative fluorescence intensity of pGSK3β. **E** Immunofluorescence detection of the expression of pGSK3β. **F** Quantitative fluorescence intensity of β-catenin. **G** Immunofluorescence detection of the expression of β-catenin. ****P<0.0001, ***P<0.001

We detected the phosphorylation level of GSK3β and the expression of β- catenin through WB (Fig. 8A) and immunofluorescence (Fig. 8E and G). The expression levels of pGSK3β (Fig. 8B) and β-catenin (Fig. 8C) were significantly reduced in the HG+SB431542 group and the immunofluorescence results were consistent (Fig. 8D and F). The result indicated that SB431542 prevented the phosphorylation of GSK3β and the expression of β-catenin in high glucose-induced RPE cells.

## Discussion

Recent studies have found that the choroidal structure and function of diabetic patients change before the development of DR [12, 13]. A high glucose environment induces the formation of CNV in the body and CNV causes subretinal fibrosis in the late stage [14, 15]. Fibrosis is primarily due to the production of extracellular matrix (ECM) in cells. During EMT, RPE cells gradually transform into fibroblast-like cells, inducing subretinal fibrosis [16]. Therefore, we focus on EMT in RPE cells in a high glucose environment in this study to provide new therapeutic targets for improving the progression of subretinal fibrosis.

Normal RPE cells present a regular polygonal structure and rely on adhesion proteins to connect closely with one another to maintain cell stability [17]. Cells tend to be fibrotic-like during EMT and the adhesion ability between cells became lower, inducing cell migration [18]. Our study found that SB431542 normalized the morphology of RPE cells and altered the expression of related protein markers, indicating that SB431542 inhibited EMT.

Mitochondria are the major source of ROS formation and mitochondrial dysfunction is considered an important cause of fibrotic diseases [8]. The ERK pathway is an important pathway that regulates mitochondrial ROS production [19]. The activation of ERK signal aggravates diabetic kidney damage by promoting the production of ROS [20]. ROS also activate ERK signaling to accelerate EMT in liver cells and promote the development of liver cancer [21]. Mitochondrial dysfunction and ERK signal activation may be mutually induced. The GSK3 β/β-catenin pathway was also associated with mitochondrial function in the survival of retinal ganglion cells [22]. SB431542 reduced the production of ROS and restored mitochondrial membrane potential in RPE cells in our study. These data indicated that SB431542 may inhibit EMT in RPE cells by maintaining mitochondrial homeostasis.

The ERK pathway regulates fibrogenesis and research has found that subretinal fibrosis is induced by activating the ERK signaling pathway [16]. GSK3β is a member of the Wnt family with compositional activity that promotes various biological events [23], which is also important in maintaining the epithelial structure of cells [24]. β-catenin is a cancer protein that is typically present in the cytoplasm and considered the central point of the Wnt/β-catenin signaling pathway, promoting the occurrence and metastasis of tumors [25]. Research has found that phosphorylated GSK3β transports β-catenin to the nucleus and phosphorylated ERK promotes the occurrence of these processes, further exacerbating the progress of EMT [26]. Our study found that SB431542 antagonized the levels of pERK1/2, pGSK3β and β-catenin induced by high glucose in RPE cells. This finding may also be related to the inhibitory effect of SB431542 on EMT.

In summary, our study determined that SB431542 may inhibit the progress of EMT by improving mitochondrial function, blocking the pERK1/2 and pGSK3β/β-catenin pathways induced by high glucose in RPE cells.

### Supplementary Information

**Additional file 1. **

## Data Availability

We promise that all data in the manuscript will be used for the first time and the data will be available.
